# Dealing with foreign cultural paradigms: A systematic review on intercultural challenges of international medical graduates

**DOI:** 10.1371/journal.pone.0181330

**Published:** 2017-07-17

**Authors:** Kerstin Michalski, Nabeel Farhan, Edith Motschall, Werner Vach, Martin Boeker

**Affiliations:** 1 Institute for Medical Biometry and Statistics, Faculty of Medicine and Medical Center – University of Freiburg, Freiburg, Germany; 2 Freiburg International Academy – University of Freiburg, Freiburg, Germany; TNO, NETHERLANDS

## Abstract

**Objectives:**

An increasing number of International Medical Graduates (IMG), who are defined to be physicians working in a country other than their country of origin and training, immigrate to Western countries. In order to ensure safe and high-quality patient care, they have to take medical and language tests. This systematic review aims to (1) collect all empiric research on intercultural communication of IMGs in medical settings, (2) identify and categorize all text passages mentioning intercultural issues in the included studies, and (3) describe the most commonly reported intercultural areas of communication of IMGs.

**Methods:**

This review was based on the PRISMA-Guidelines for systematic reviews. We conducted a broad and systematic electronic literature search for empiric research in the following databases: MEDLINE, BIOSIS Citation Index, BIOSIS Previews, KCI-Korean Journal Database and SciELO Citation Index. The search results were synthesized and analyzed with the aid of coding systems. These coding systems were based on textual analysis and derived from the themes and topics of the results and discussion sections from the included studies. A quality assessment was performed, comparing the studies with their corresponding checklist (COREQ or STROBE). Textual results of the studies were extracted and categorized.

**Results:**

Among 10,630 search results, 47 studies were identified for analysis. 31 studies were qualitative, 12 quantitative and 4 studies used mixed methods. The quality assessment revealed a low level of quality of the studies in general. The following intercultural problems were identified: IMGs were not familiar with shared decision-making and lower hierarchies in the health care system in general. They had difficulties with patient-centered care, the subtleties of the foreign language and with the organizational structures of the new health care system. In addition, they described the medical education in their home countries as science-oriented, without focusing on psychosocial aspects.

**Conclusion:**

There is a need for a better training of IMGs on culture-related and not culture-related topics in the new workplace country. The topics that emerged in this review constitute a basis for developing these courses. Further empiric research is needed to describe the findings of this review more precisely and should be in accordance with the existing reporting guidelines.

## Introduction

International Medical Graduates (IMG) have become an important workforce as ‘foreign doctors’ in the health systems of most developed countries due to a shortage of health professionals in aging societies. Although meeting urgent demands, this immigration of trained physicians comes for a certain price and new difficulties arise during the immigration process. E.g., due to the lack of physicians in Germany [1], more and more International Medical Graduates (IMGs) immigrate to fill in these gaps particularly in economically weak regions [2]. In 2016 they already made up 11% of the German medical workforce, with the majority coming from Southeastern Europe and Syria [3]. In English-speaking countries such as the USA, Canada, Australia and the United Kingdom, IMGs even constitute of 24% and 33% [4] of the medical workforce. These foreign physicians mostly emigrate from Asian and Middle Eastern regions or from the respective neighboring countries of the receiving countries (e.g. Canadians who emigrate to the US) [5]. In all these countries, IMGs must pass medical equivalency exams and attest high language proficiency before they are provisionally or fully licensed to practice. Despite this quality assurance, many communication problems with patients and colleagues are reported, assuming that these difficulties are caused by cultural differences and not by language problems or a lack of medical competence [6–8]. Nevertheless, cultural differences are a widely neglected topic both in equivalency exams or in empirical research with regard to IMGs [9–11]. Consequently, a detailed description of these intercultural communication problems is necessary for developing courses or exams capable of improving the situation. To our knowledge, the Australian review from Pilotto et al. [12] is the only study specifically addressing communication problems in an intercultural context. In their article, the authors provide suggestions to Australian physicians for better training of their international colleagues. The authors mention some cultural challenges without explicitly addressing these issues. However, this article was not meant to be sufficient for a detailed description of intercultural problems concerning IMGs, since it was short in this respect and only treated a limited number of aspects of intercultural communication.

Hence, there still is a need for a detailed and systematic description of intercultural communication by International Medical Graduates with patients, their families and team members. In order to address these topics, this systematic review has the objectives to (1) collect all empiric research on intercultural communication of IMGs in medical settings, (2) identify and categorize all text passages mentioning intercultural issues in the included studies, and (3) describe the most commonly reported intercultural areas of communication of IMGs.

## Methods

This systematic review adheres to the reporting guidelines and criteria set in Preferred Reporting Items for Systematic Reviews (PRISMA) [13]. A protocol for this systematic review has been compiled but publishing attempts were not undertaken.

### Search strategy

Empirical studies were searched, in which the observed population consisted of International Medical Graduates (IMGs) who were defined to be physicians working in a country other than their country of origin and training. Not relevant in this context was whether these studies used qualitative or quantitative methods for analyzing intercultural communication situations of these foreign physicians. The literature search included papers from the earliest time available in specified databases up to 31/07/2014. In 2017 a search update was conducted and articles up to 05/04/2017 were searched. Papers in languages other than English or German were excluded. The first search as well as the updated search followed the standards of the Cochrane Collaboration [14] and covered several databases: MEDLINE (via OVID SP and Web of Science), BIOSIS Citation Index, BIOSIS Previews, KCI-Korean Journal Database, SciELO Citation Index (via Web of Science). A Medical Subject Heading (MeSH) keyword search using the MeSH *Foreign Medical Graduates/* was conducted via OVID SP. Web of Science was searched for text words covering all other databases, using different and truncated designations for International Medical Graduate and combining the individual words with a proximity operator. For the complete search expressions, please refer to S1 Text.

### Study selection

CITAVI 4.4 from Swiss Academic Software was used to organize the results of the individual database searches. Duplicates were removed before the results from all databases were compiled. After deleting all new duplicates, articles were screened by title and abstract (KM). A second researcher (NF) screened the 500 most recent records from the first search and an inter-rater reliability was calculated for these 500 studies. Disagreements were resolved by consensus between these two reviewers. Full texts of all selected studies were evaluated for eligibility in an additional step (KM). Articles causing uncertainty as to their inclusion were evaluated by a third researcher (MB). Articles were considered for review if they consisted of empirical studies reporting quantitative or qualitative data of the intercultural context of IMGs. For a detailed list of inclusion and exclusion criteria please refer to S2 and S3 Text.

### Data extraction

Data was extracted from relevant text passages if it contained information on the objective of the study, demographics and profession of study participants, the intercultural setting of the study, study methodology, or results of the study. The following data was extracted and listed in separate tables for qualitative and quantitative studies: detailed description of the study participants (number, region of origin of the IMGs, specialist field of the IMGs and training status of the IMGs); detailed description of the methods while differentiating between qualitative (interviews, focus groups, author notes or evaluation of a video-taped conversation) and quantitative (supervisor rating scale or questionnaire with number and origin of items and instruments) methods; detailed description of the intercultural setting (year of publication, country of the study, ambulatory or hospital setting); question and aim of the study, the results of the study rendered as text (qualitative and quantitative) or data (quantitative).

### Category systems and coding

For qualitative studies, a two-level hierarchical category system was derived from the themes and topics of the results and discussion sections of the included studies based on text analysis. The category system was exhaustive with respect to themes and topics in relation with foreign doctors: all themes and topics of the included studies regarding IMGs could be represented by the category system. The category system was derived, discussed and consented between the researchers (KM, NF, and MB). The intended meaning of the categories was described in short text definitions. A more detailed subtype of a main-category is a sub-category and is ordered hierarchically below the corresponding main-category. The following main-categories were identified in the qualitative studies: 1.1 Communication with patients, 1.2 Communication with relatives, 1.3 Communication with native physicians, 1.4 Communication with other health professionals, 1.5 Nonverbal communication 1.6 Communication unspecified, 2.1 Health care system, 3.1 Language, 4.1 Status of physicians, 5.1 Origin of IMGs, 6.1 Immigration, 7.1 Racism/discrimination and 8.1 Gender issues. The main-categories and their descriptions are shown in Table 1. A complete version of the category system including the sub-categories is provided in S1 Table. Besides the main-category system a type of statement was defined which can have the following values: T-1 = Problem, T-2 = Improvement opportunity, T-3 = Difference or T-4 = Positive judgment/attitude.

**Table 1:** Main-categories of the qualitative coding system.

Code	Main-category	Description
A-1.1	Communication with patients	Way of patient treatment (patient-centered or directive), information and medication, duration of treatment, emotional support, the decision-making style, patient compliance and hierarchy in the physician-patient relationship
A-1.2	Communication with relatives	Involvement of the patient’s family in information, decision-making, treatment and care
A-1.3	Communication with native physicians	Mentions about patient presentation, supervisor support and hierarchy among physicians
A-1.4	Communication with other health professionals	Allocation of tasks and hierarchy in an interdisciplinary team
A-1.5	Nonverbal communication	All types of nonverbal communication
A-1.6	Communication unspecified	Communication in general without specifying the counterpart
A-2.1	Health care system	Patient documentation and the organizational, economic and legal parameters of a health care system
A-3.1	Language	Basic language skills and more specific abilities in the use and the comprehension of dialect, small-talk, common speech, accent and medical terminology
A-4.1	Status of physicians	The general status of doctors in society, in the health care system, in the physician-patient relationship and in an interdisciplinary team
A-5.1	Origin of the IMGs	The cultural and educational background of foreign physicians
A-6.1	Immigration	The cultural, organizational and work-related issues of the immigration process of IMGs
A-7.1	Racism/discrimination	All kinds of racism or discrimination against foreign physicians
A-8.1	Gender issues	All aspects concerning the differences of sexes

For the thematic grouping of quantitative studies, a category system based on the questions and instruments of the quantitative studies was developed and agreed on by the authors KM, NF, WV and MB. A complete version of the category system is provided in S2 Table. In order to allow for contentwise comparison of the very heterogeneous quantitative studies, a second two-level hierarchical category system based on the textual results of the quantitative studies was developed and agreed on by the authors KM, NF, and MB. It consisted of the main-categories shown in Table 2. A complete version of the category system is provided in S3 Table.

**Table 2:** Main-categories of the quantitative coding system.

Code	Main-category	Description
C-1.1	Context—work	Work related stress, resources and well-being
C-1.2	Context—colleagues/team	Differentiation between peers, supervisors and other health professionals
C-1.3	Context—patients/relatives	All issues about patients and their families
C-2.1	Communication	Differentiation between the individual communication between two physicians from the communication in a group of physicians
C-3.1	Health care system	Organizational structures and the billing system of a health care system
C-4.1	Clinical skills	All issues concerning the clinical/practical skills of IMGs

Text passages were marked for a category and a type (coded) if the topic or theme of the text passage corresponded with the meaning and type of the category. Thus, each of these coded text passages [15] mentions the category or categories for which it has been marked and which refers to it. Therefore, it is called a ‘mention’ in the sense that the text passage ‘is about’ the corresponding category and is of a specific type that constitutes an assessment of the statement. E.g. ‘IMG physicians however reported that carrying out small talk was a key difficulty with their patients (A-1.1 Communication—Communication with patients, A-3.1.6 Language—Small talk or humor, T-1 Type—Problem) […] Most IMG interns interviewed in the study reported dissatisfaction with the use of abbreviations by medical teams because they are still not acculturated enough to learn the medical lingo’ (3.1.4 Language—Medical terminology, T-1 Type—Problem). Text extracted from Jain & Krieger (2011). The extent of the mentions was marked exactly in the text of the sources. The extent of the mentions could overlap other mentions. No special software for qualitative annotation and analysis has been employed. In the case of non-matching categories between coders for the same mentions, agreement was reached by discussion.

By coding mentions with more than one category, the categories could generally be used compositionally (see example above). In this context, the type could be used to encode the assessments of the authors/probands about a topic which is expressed in the mention. Thus, the type denotes qualifying statements on the topic of the mention, e.g. a mention coded with ‘T-1 Type—Problem’ has the meaning that a *problem* in the results for the topics/categories of the mention was expressed.

With both category systems, all included quantitative and qualitative studies were coded by two authors (KM and NF for the first version of this review and KM and MB for the updated search results) as described above.

### Risk of bias

The quality of the studies using qualitative methods was assessed by the 32-item consolidated criteria for reporting qualitative research (COREQ) checklist [16]. The quantitative studies were processed similarly, using the 22-item strengthening the reporting of observational studies in epidemiology (STROBE) checklist [17]. Additionally, the origin and validated source (if applicable) of the instruments were noted.

### Synthesis of results

The mentions of the qualitative categorization of the qualitative and quantitative studies were counted and sums were tabularized for the main-categories. The frequencies of mentions in the studies were retrieved from these tables. Furthermore, detailed conclusions were derived from the analysis of the frequencies in the sub-categories of the qualitative studies. Since most quantitative studies did not include a detailed description of intercultural issues, a further description of intercultural communication of IMGs could not be generated by means of these studies.

## Results

### Study selection

The first electronic database search identified 9,688 records. After removing duplicates separately for the two search interfaces OVID SP and Web of Science, the total search comprised 6,632 records. 6,595 records were excluded based on title and abstract screening because these articles did not conform to the inclusion criteria (see list S2 Text). Of the remaining 37 full texts, 7 full texts were excluded for missing aspects of culture or communication or the fact that some studies were not empirical studies. Finally, 30 studies were included in the first version of this review. The selection process of the first search is illustrated in S1 Fig. The second electronic database search identified 942 articles. Again, duplicates were removed separately for the two search interfaces and 774 records were screened based on title and abstract. 754 articles were excluded, because they did not conform to the inclusion criteria. Of the remaining 20 full texts, 6 full texts were excluded because they were not published in English or German, the observed population was not physicians or cultural aspects were missing. In addition, 3 articles were identified by hand search. Including 17 new articles of the updated search, a total of 47 papers were reviewed. 31 of them were qualitative studies, 12 were quantitative and 4 studies used mixed methods (for a listing see Table 3).

**Table 3:** Studies included in the review.

Qualitative	Chen et al. (2010) [19], Curran et al. (2008) [20], Dahm (2011) [21], Dahm (2011) [22], Dahm et al. (2015) [23], Diaz et al. (2011) [24], Dorgan et al. (2009) [6], Fiscella et al. (2000) [25], Huijskens et al. (2010) [26], Jain et al. (2011) [27], Klingler et al. (2016) [28], Legido-Quigley et al. (2015) [29], Lockyer et al. (2007) [30], Lockyer et al. (2010) [31], Mahajan et al. (2007) [32], McDonnel et al. (2008) [33], McGrath et al. (2011) [34], McGrath et al. (2012) [35], Morrow et al. (2013) [36], Osta et al. (2016) [37], Rao et al. (2013) [38], Searight et al (2006) [39], Skjeggestad et al. (2017) [40], Slowther et al. (2012) [41], Sommer et al. (2012) [42], Teodorescu et al. (2013) [43], Triscott et al. (2016) [44], Verma et al. (2016) [45], Warwick et al. (2014) [46], Woodward-Kron et al. (2015) [47], Yates et al. (2016) [48]
Quantitative	Aalto et al. (2014) [49], Fernandez-Pol et al. (1989) [50], Harding et al. (2010) [51] Kuusio et al. (2013) [52], Kwon et al. (1984) [53], Lillis et al. (2014) [54], Myerholz (2014) [55], Narasimhan et al. (2006) [56], Pantenburg et al. (2016) [57], Rolfe et al. (1994) [58], Sullivan et al. (2001) [59], Zulla et al. (2008) [60]
Mixed methods	Gasiorek et al. (2012) [61], Hall et al. (2004) [8], Sockalingam et al. (2014) [62], Terry et al. (2014) [63]

**Fig 1:**
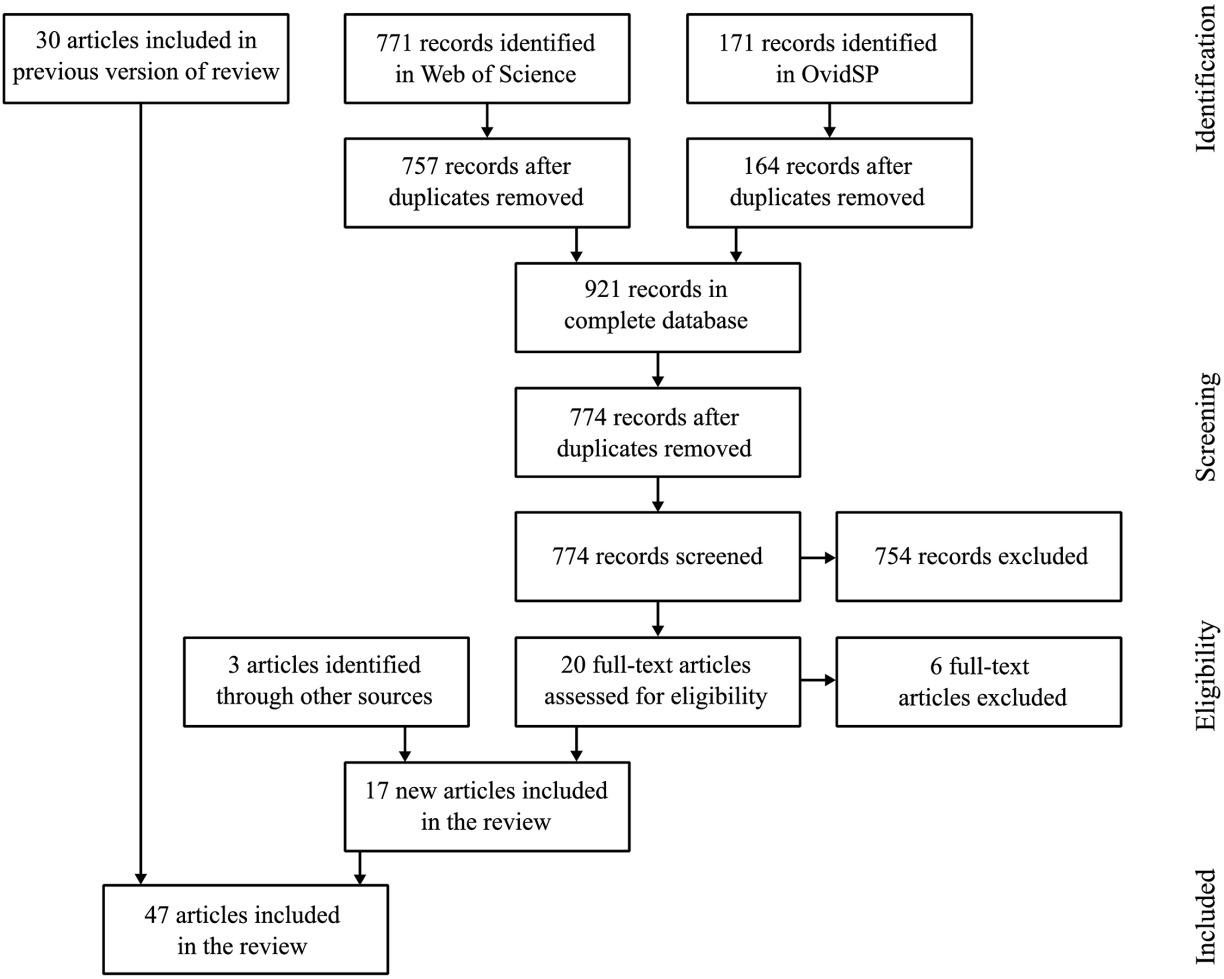
PRISMA Flow diagram of study selection.

A brief summary of the results of the studies and the country of origin of the IMGs is provided in S4 Table for the qualitative and in S5 Table for the quantitative studies. The results of the inter-rater comparison of the 500 most recent records of the first search are shown in Table 4. An accordance with a *κ* = 0.5658 was found, which was considered a good inter-rater reliability. The complete selection process is illustrated in the adapted PRISMA Flow diagram Fig 1 according to Stovold et al. [18].

**Table 4:** Inter-rater comparison of the 500 most recent records of the first search. NF = Nabeel Farhan. KM = Kerstin Michalski.

	Inclusion KM	Exclusion KM
Inclusion NF	4	5
Exclusion NF	1	490

### Study characteristics

Most of study participants in the qualitative and mixed methods studies (*n* = 35) were IMGs (1,004 out of 1,183) and the majority of these studies showed solely the perception of the IMGs and not the point of view of their colleagues (23 of 35 studies). The foreign physicians participating in the qualitative studies mostly came from Southern Asia (especially India), the Middle East, and Eastern Europe. They often worked in ambulatory or hospital primary care settings as general practitioners or internal specialists. Only few studies noted the training status of their participants (12 of 35 studies). The qualitative studies were conducted between the years 1997 and 2017, most of them being published after the year 2010 (29 of 35). The majority was conducted in English-speaking countries such as North America (13 of 35), Australia (10 of 35), and the United Kingdom (6 of 35). The remaining articles (6 of 35) were published in other European countries. Most of the qualitative studies used (telephone) interviews (27 of 35), or additionally or solely focus groups (10 of 35). Some authors evaluated a video-recorded consultation of IMGS (4 of 35).

In comparison with the qualitative studies, the quantitative studies had other characteristics: Most of the study participants were native health personnel, mainly physicians or patients whereas IMGs were the minority (1,682 of 11,748 study participants). The quantitative articles were published between the years 1984 and 2016, but mostly after the year 2000 (9 of 12). They were performed in North America (5 of 12), Oceania (4 of 12) and different European countries (3 of 12). The foreign doctors often worked as general practitioners or internists, but also as surgeons, predominantly in a hospital setting. Astonishingly, the region of their origin (9 of 12) and their training status (7 of 12) was mostly unknown. The authors of the quantitative studies almost exclusively used (paper and online) questionnaires (10 of 12). Only two studies used a supervisor rating scale.

### Methodical quality of the studies

In the risk of bias evaluation of the studies by means of the COREQ- [16] and the STROBE-Checklist [17], most studies reached comparatively few points on the respective scales indicating a low methodical level of quality. On average, the qualitative and the corresponding part of the mixed methods studies achieved 18 of 32 points on the COREQ-Checklist, with a range of 11.5 to 24 points. The quantitative and the mixed-methods studies achieved a mean value of 15 of 22 points on the STROBE-Checklist with a minimum of 8,5 and a maximum of 20 points. In addition, a high heterogeneity in the instruments and items of the quantitative studies was found, with only two studies using similar methods. Furthermore, 6 of 16 studies used invalidated instruments and items.

### Intercultural differences

In order to provide insight into the assessment of topics by participants of the qualitative studies, Table 5 shows counts of mentions in the main-categories vs. the four assessment types. The total frequencies of the mentions in the qualitative studies are shown in the last column of Table 5.

**Table 5:** Frequency of mentions by topic in the qualitative (*n* = 31) and mixed methods studies (*n* = 4; *n*_sum_ = 35) and their assessment types. Percentage in parenthesis relative to the row sum.

Topic	T-1		T-2		T-3		T-4		Sum	Total
	*n*	[%]	*n*	[%]	*n*	[%]	*n*	[%]	*n*	[%]
A-1.1 Communication with patients	33	[42]	4	[5]	30	[39]	11	[14]	78	21
A-1.2 Communication with relatives	3	[20]	0	[0]	12	[80]	0	[0]	15	4
A-1.3 Communication with native physicians	12	[46]	0	[0]	9	[35]	5	[19]	26	7
A-1.4 Communication with other health professionals	13	[59]	2	[9]	6	[27]	1	[5]	22	6
A-1.5 Nonverbal communication	4	[50]	2	[25]	1	[12, 5]	1	[12, 5]	8	2
A-1.6 Communication (unspecific)	4	[50]	2	[25]	0	[0]	2	[25]	8	2
A-2.1 Health care system	24	[52]	11	[24]	9	[20]	2	[4]	46	12
A-3.1 Language	51	[76]	9	[13]	2	[3]	5	[8]	67	18
A-4.1 Status of physicians	10	[34]	1	[3]	19	[63]	0	[0]	30	8
A-5.1 Origin of the IMGs	6	[37, 5]	1	[6]	6	[37, 5]	3	[19]	16	4
A-6.1 Immigration	25	[47]	7	[13]	10	[19]	11	[21]	53	14
A-7.1 Racism/Discrimination towards IMGs	4	[80]	0	[0]	0	[0]	1	[20]	5	1
A-8.1 Gender issues	3	[75]	0	[0]	1	[25]	0	[0]	4	1
All categories	192	[51]	39	[10]	105	[28]	42	[11]	378	100

Type: T-1 = Problem, T-2 = Improvement opportunity, T-3 = Difference, T-4 = Positive.

Overall ‘communication with different counterparts’ was the topic mentioned the most often in the qualitative studies (42% in total), whereas ‘language’ (18%), ‘immigration’ (14%), ‘health care system’ (12%) and ‘status of physicians’ (8%) were topics also frequently mentioned. It should be noted that these topics (except of ‘immigration’) were part of the inclusion criteria.‘Origin of the IMGs’, ‘racism/discrimination’ and ‘gender issues’ were not mentioned as often. It is interesting that ‘immigration’ achieved more mentions (14%) than ‘status of physicians’ (8%), although it was not an eligibility criterion. ‘Racism/discrimination’ and ‘gender issues’ were very rare topics. Most mentions were assessed or rather described as ‘problems’ (51%). Assessments as ‘differences’ (28%) were less often. Positively assessed mentions or ‘improvement opportunities’ (10%) were infrequent (11%). It is remarkable that mentions in ‘health care system’ and ‘language’ were almost exclusively negatively connoted, describing many ‘problems’ and ‘improvement possibilities’, whereas mentions in ‘communication with different counterparts’ also often described differences.

**Fig 2:**
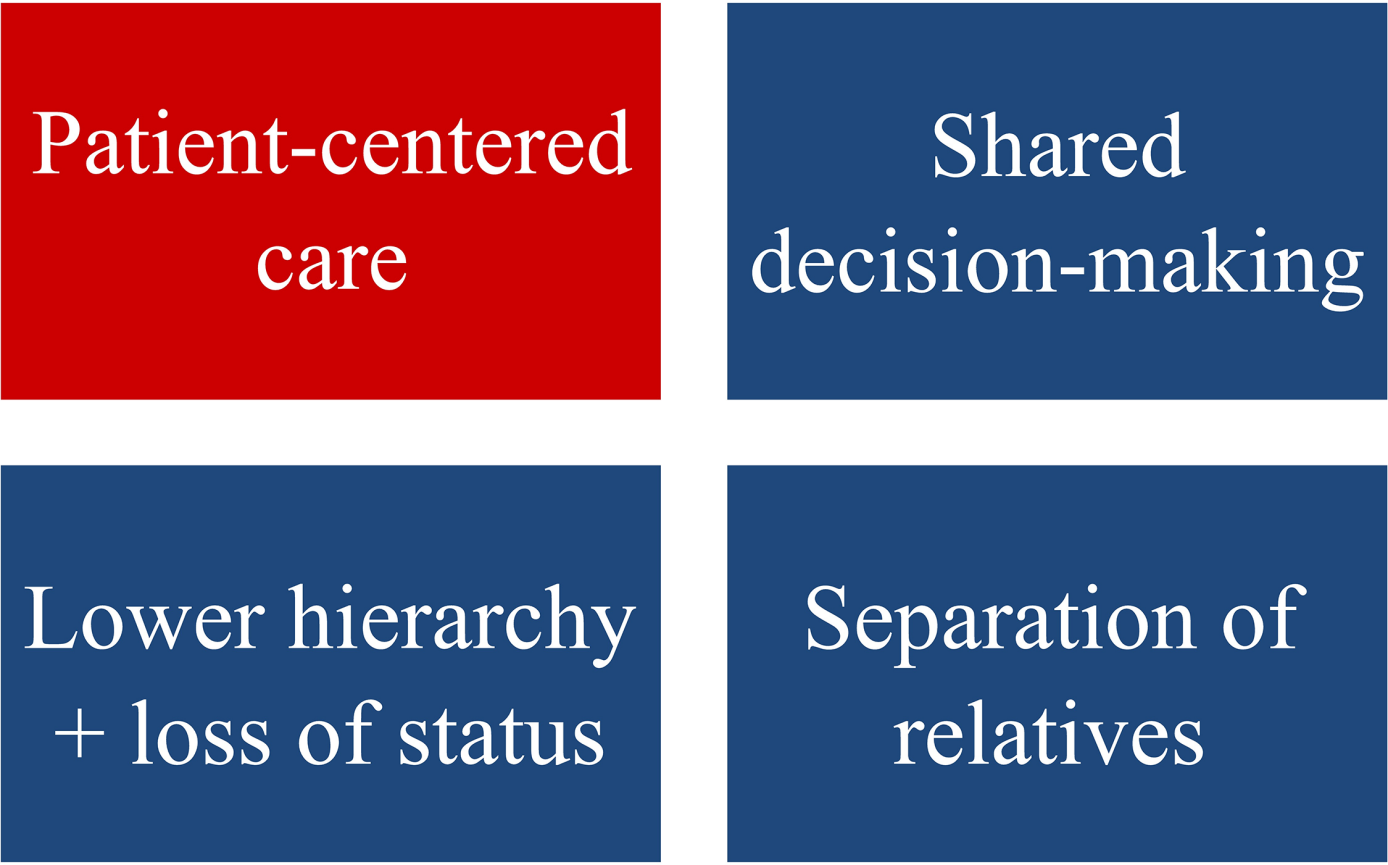
Culture-related results of the qualitative and mixed methods studies. The red color indicates problems for the IMGs with this issue. The blue color indicates differences between the home and the new culture of the IMGs.

The detailed results over all sub-categories with a numbering of the respective studies are provided in S6 Table. Based on these results, culture-related influences can be summarized as follows and are depicted in Fig 2. Corresponding citations from original studies are provided to illustrate the individual items:

1. IMGs had difficulties with the concept of patient-centered care and were not familiar with the concept of shared decision-making before they had immigrated.
  ‘(…) it emerged that IMGs encounter several challenges with adapting to PCC.’ [21].
  ‘Residents described many differences between U.S. family medicine and their previous training experiences with regard to the physician-patient relationship. These differences reflected a consumer-oriented versus a paternalistic view of patient care. For example, patients receiving care in the United States were perceived as more inquisitive and active in medical decision making (…)’ [39].
2. They were not used to separate the relatives from the information and treatment process, since in their (mainly Middle Eastern and Asian) home culture the family of the patient was addressed before the patient himself.
  ‘It was more common in their experience to discuss diagnosis, treatment and care plans with families, particularly male family members, than directly with the patient.’ [8].
3. They described a relatively low hierarchical distance to their supervisors, as well as a lower hierarchy with patients and with other health professionals.
  ‘Most of the participants described coming from, and being trained in, cultures in which physicians were regarded with a great deal of respect, and the physician-patient relationship was more vertical—that is, the physician had the authority in the relationship and, therefore, made the decisions. Several IMGs reported that patients in their cultures of origin believed that physicians were godlike.’ [6].
  ‘The organization of the Dutch health care system is less hierarchical than the environments in which most IMGs were first trained.’ [26].
4. Overall they described a loss of status in society and the clinical environment.
  ‘Many of the IMGs came from countries where the doctors’ status was high and neither patients nor their family questioned the doctors’ authority.’ [35].

**Fig 3:**
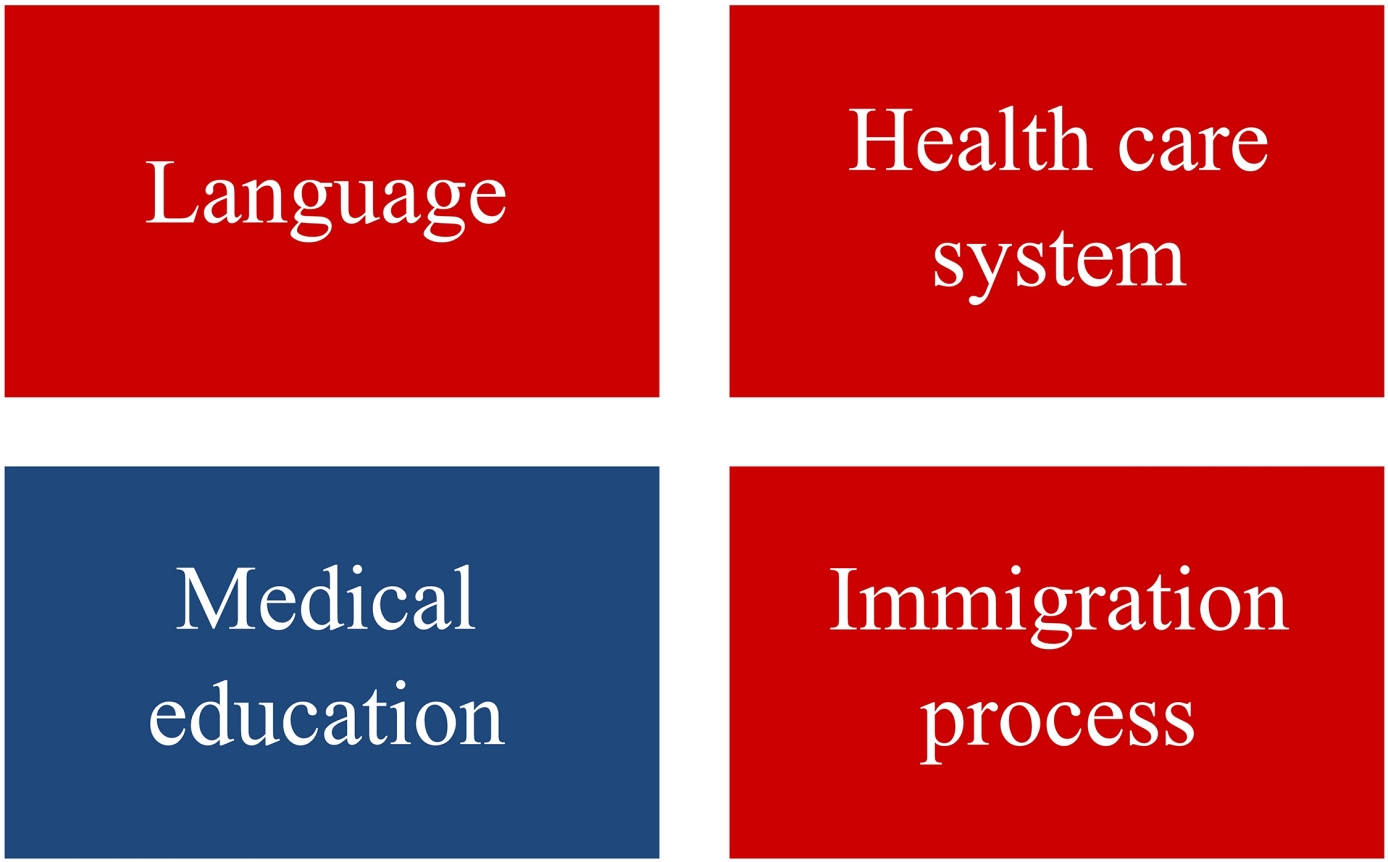
Non-culture-related results of the qualitative and mixed methods studies. The red color indicates problems for the IMGs with these issues. The blue color indicates that this issue was different in the home country of the IMGs.

Non-culture-related influences can be summarized as follows and are depicted in Fig 3:

1. IMGs felt relatively confident in the basic use of the foreign language, but they had difficulties with the use and understanding of common language (incl. dialect), their accent and the use of medical terminology.
  ‘Even when MMPs (Mobile Medical Professionals) have a reasonable mastery of the standard language of their new country, they continue to have problems understanding and using local and regional dialects in interaction with both colleagues and patients.’ [61].
  ‘IMGs also often experienced difficulties in distinguishing everyday language and medical terminology (…)’ [22].
2. They talked about problems in dealing with the specific rules and the organizational structures of the new health care system.
  ‘Finally, respondents were unaccustomed to the system of checks and balances in US healthcare and physicians’ sensitivity to potential litigation.’ [19].
3. They described their medical education as science-oriented, focusing on the development and treatment of diseases rather than on the psychosocial aspects of the medical profession such as responding to the patients’ fears and worries concerning their medical attendance.
  ‘Participants often mentioned that they came from cultures that highly value education; however, in those cultures, ‘education’ tended to be defined in a specific way as a focus on the hard sciences.’ [6].
4. The foreign physicians also faced usual difficulties of immigration in a new country. Especially general cultural and organizational obstacles were described.
  ‘Aspects of practice which IMGs highlighted as difficult to understand (…)(:) The culture and system of UK general practice—which they generally felt was a complete unknown to them, even after successful selection into the career path.’ [46].
  ‘Reported barriers included difficulties in accessing information on complementary medical education and lack of (financial) support.’ [26].

**Fig 4:**
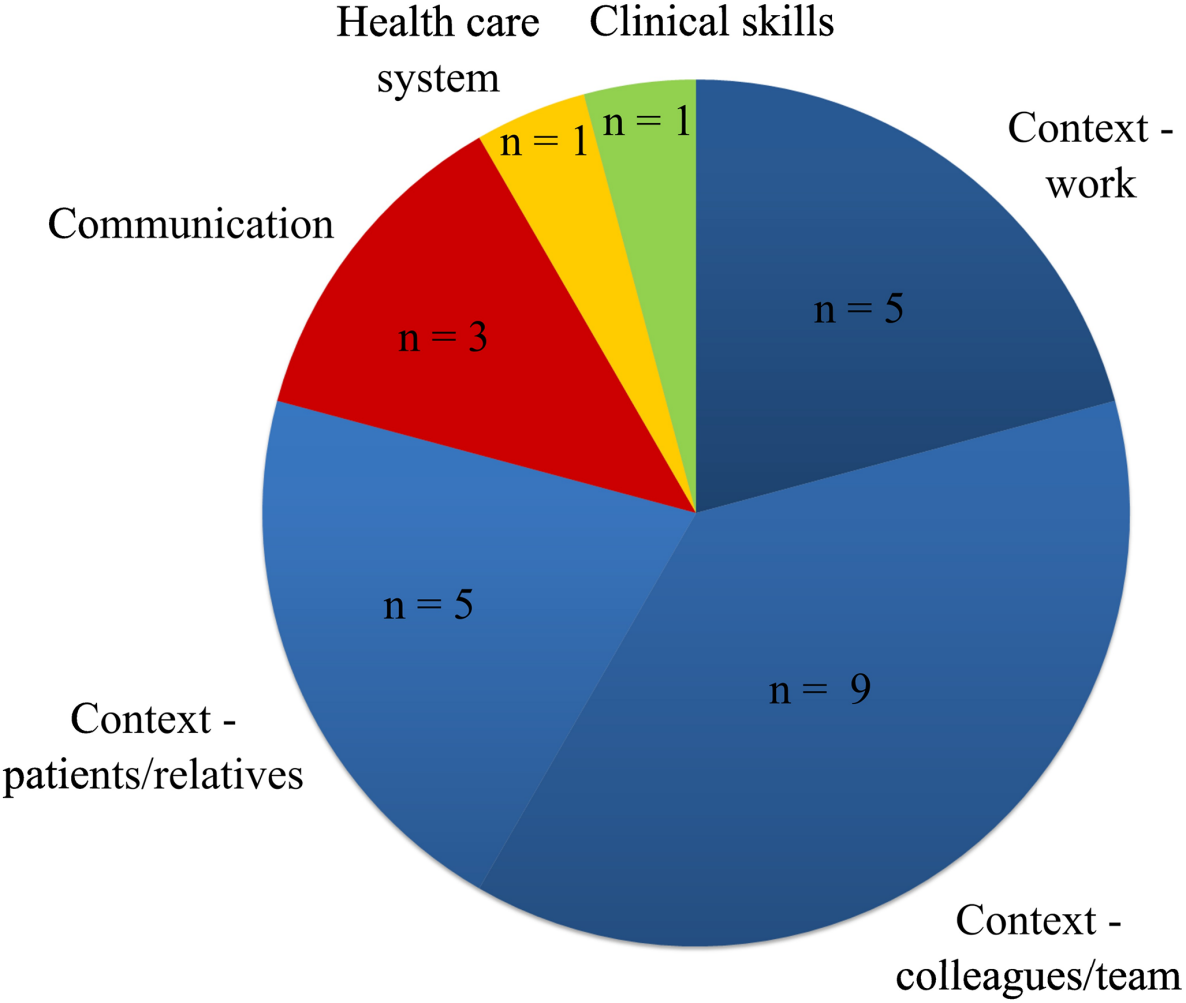
Absolute frequency of the survey aims in the quantitative (*n* = 12) and mixed methods studies (*n* = 4; *n*_sum_ = 16).

The quantitative studies varied largely in aims and purposes. Except for the main-category ‘context—colleagues/team’ which was found in 9 of 16 studies, the study-questions and instruments varied considerably (Fig 4).

The frequency of mentions based on the textual results of the quantitative studies was analyzed as a second step to reveal congruences in the results of these studies.Table 6 shows the counts of mentions in the main-categories vs. the four types for the quantitative studies. The total frequencies of the mentions in the quantitative studies are shown in the last column of Table 6

**Table 6:** Mentions in the quantitative (*n* = 12) and mixed methods studies (*n* = 4; *n*_sum_ = 16) and their assessment types. Percentage in parenthesis relative to the row sum.

Topic	T-1		T-2		T-3		T-4		Sum	Total
	*n*	[%]	*n*	[%]	*n*	[%]	*n*	[%]	*n*	[%]
C-1.1 Work	2	[22]	0	[0]	6	[67]	1	[11]	9	17
C-1.2 Colleagues/team	2	[18]	1	[9]	6	[55]	2	[18]	11	21
C-1.3 Patients/relatives	3	[25]	1	[8]	7	[59]	1	[8]	12	23
C-2.1 Communication	1	[17]	1	[17]	3	[50]	1	[17]	6	11
C-3.1 Health care system	5	[46]	2	[18]	3	[27]	1	[9]	11	21
C-4.1 Clinical skills	3	[75]	0	[0]	1	[25]	0	[0]	4	7
All categories	16	[30]	5	[10]	26	[49]	6	[11]	53	100

Type: T-1 = Problem, T-2 = Improvement opportunity, T-3 = Difference, T-4 = Positive.

The quantitative studies focus slightly on the categories ‘context—colleagues/team’, ‘context—patients/relatives’, and ‘health care system’ related to the results in the qualitative studies. Results for the quantitative studies are reported on an individual level because of their large heterogeneity and were not further aggregated. Most quantitative studies did not include a detailed description of intercultural issues, so that a further description of intercultural communication of IMGs could not be generated. The assessment types showed a clear emphasis on ‘differences’ (49%) which is most likely due to the design of included studies comparing foreign with native physicians or other health professionals (12 of 16 studies).

## Discussion

This systematic review included forty-seven studies about intercultural communication of International Medical Graduates. To our knowledge, it is the first systematic approach with this objective solely based on empiric research. Some commonly mentioned culture-related and non-culture-related influences on the IMGs were identified. The synthesis of the studies revealed that IMGs were not familiar with the concept of shared decision-making and lower hierarchies in the health care system in general. They had difficulties with patient-centered care, the subtleties of the foreign language and with the organizational structures of the new health care system. Furthermore, they described the medical education in their home countries as science-oriented, without focusing on psychosocial aspects.

Regarding the literature on intercultural problems, the work of Pilotto et al. (2007) [12] deserves special consideration, as they wrote about difficulties for IMGs in dealing with an equitable doctor-patient relationship, patient-centered care and the loss of status. These topics also emerged in this review but were—except for patient-centered care—described as differences of the home and the new culture and not as problems. In addition, difficulties for the foreign physicians with the concept of shared decision-making as described by Khan et al. (2014) [10] were not found. The results of this review indicate that the IMGs did not consider this concept as problematic. Pilotto et al. (2007) [12] and Khan et al. (2014) [10] both described the medical education in the domestic countries of the IMGs as directive and authoritarian. These findings could not be reproduced directly, but evidence was found that IMGs were educated science-oriented, without focusing on the psychosocial aspects of the medical profession. Furthermore, the difficulties of IMGs with the foreign language, which were already found by Pilotto et al. (2007) [12], could be described in more detail in this review: foreign doctors have problems with the use and understanding of common language, their own accent, the different dialects as well as the use of medical terminology and not only with the basic language use. Difficulties with the foreign language were also described in a recent letter by Kramer (2015) [64] who also noted the different hierarchical expectations of International Medical Graduates (mostly coming from Asia) and their Western supervisors, which correspond to findings of this review. The description of general problems during the immigration process by Kalra et al. (2012) [9] could also be reproduced. Kaafarani [65] mentioned 2009 in a narrative review discrimination as a source of problems for the IMGs beside cultural and linguistic challenges. This discrimination was due to structural difficulties for foreign physicians but also included overt and subtle forms of discrimination. In the studies included in this review, discrimination did not appear as a focus, though it was mentioned.

To our knowledge, some topics concerning intercultural communication of foreign doctors were reported in a systematic review for the first time. The studies included in this review indicate that it was unknown for IMGs to separate the family of the patient during the information and treatment process of the patient. Furthermore, problems for the foreign doctors with the new health care system and its specific organizational structure were found. Racism, discrimination and gender issues hardly emerged in the included studies. This could be due to hesitations of the IMGs to talk about this topic or because it was not relevant to them.

### Limitations

Not all available databases were searched in this review. Due to limited resources, the search was concentrated on the most relevant databases for medical and health professionals, including MEDLINE and other databases accessible via the common search interface of Web of Science. Furthermore, a broad search strategy was conducted in the included databases rather than searching additional databases. Part of the search results was screened by only one researcher. However, a good inter-rater agreement was found and inclusion of ‘difficult’ studies was discussed by at least two researchers.

Few quantitative studies were included despite a broad and systematic electronic literature search in several databases. The included quantitative studies were of low methodical quality and lacked precise descriptions of intercultural communication. Hence, the majority of the conclusions were retrieved from qualitative studies, which had an equally low level of quality and mostly presented the perception of the IMGs and rarely the point of view of their native colleagues.

Only the reported topics in the included studies were synthesized. Consequently, the authors of the studies decided whether an issue was considered relevant. This should be noted especially when analyzing the quantitative studies, where the study participants could only answer predetermined questions. Furthermore, the questions of the quantitative surveys were not based on the topics that emerged in the qualitative studies, because many of the quantitative studies were published prior to the qualitative articles.

In our review the observed IMG population was handled as one homogeneous population, but in reality it is very heterogeneous. Stratification by factors like the time already spent in the new health system or the cultural background would be very informative, but only few studies included such a differentiation.

This review did not include studies in languages other than English or German and thus may under-represent articles from countries which publish in other languages.

Finally, it cannot be excluded in such a review that the results also reflect certain opinions or attitudes common among researchers conducting such studies, which may have influenced their choice of instruments and their analysis. For example, it might be a little surprising that none of the studies highlighted the bilateral responsibility that also includes the duties and responsibilities of the new health system.

### Implications for future research

Future research on intercultural communication of IMGs should be reported according to the quality standards of the COREQ-Checklist [16] for qualitative articles or the STROBE-Checklist [17] for quantitative surveys. Authors should also include the perception of the native physicians or health professionals, as well as the opinion of the patients. In addition, they should also focus on the bilateral responsibility of an effective immigration process. Quantitative studies need a consensus about the instruments and items that are used to make a future quantitative synthesis possible. More studies from non-English-speaking countries would be of large value. Finally, future research should also focus on nonverbal communication, an important sub-domain of intercultural communication.

### Conclusion

This study systematically searched and analyzed empiric research about intercultural communication of International Medical Graduates and reported the results according to the PRISMA-Protocol [13]. 47 eligible articles (31 qualitative, 12 quantitative, 4 mixed-methods studies) out of 10,630 search results were identified.

The main findings indicate that IMGs were unfamiliar with the concept of shared decision-making and the separation of relatives from the information and treatment process. The concept of patient-centered care was described to be difficult to them. Furthermore, IMGs described a loss of status and flat hierarchies to supervisors, to other health professionals and to patients compared to their domestic countries in which they were trained. In addition, non-culture-related difficulties for the IMGs with the new health care system and with the subtleties of the foreign language were found. The education of the IMGs was described as science-oriented without focusing on psychosocial aspects. At last, the findings suggest general problems for IMGs during the immigration process.

This systematic review constitutes a basis for future research on intercultural communication of IMGs. Further, it indicates the need for training of foreign physicians on culture-related and non-culture-related issues of the new workplace country. Since an effective immigration process is a two-sided process, the hosting health systems should provide introductory and training material for arriving IMGs on which they depend on to fill their gaps. The results of this review can give a direction for the development of appropriate curricula and material for the IMGs and their colleagues to achieve a better understanding of the intercultural areas of communication of both sides.

## Supporting information

S1 Fig PRISMA Flow diagramm of the first search. (PDF)

S1 Text Search strategy via Web of Science. (PDF)

S2 Text Inclusion criteria. (PDF)

S3 Text Exclusion criteria. (PDF)

S1 Checklist PRISMA checklist. (PDF)

S1 Table Category system of the qualitative studies. (PDF)

S2 Table Category system based on the questions and instruments of the quantitative studies. (PDF)

S3 Table Category system based on the textual results of the quantitative studies. (PDF)

S4 Table Qualitative studies included in the review. (PDF)

S5 Table Quantitative studies included in the review. (PDF)

S6 Table Results of the qualitative studies. (PDF)
