# Supplementary material for: Dealing with foreign cultural paradigms: A systematic review on intercultural challenges of international medical graduates
Source: PLoS One. 2017 Jul 17;12(7):e0181330. doi: 10.1371/journal.pone.0181330 (PMC5513557; doi:10.1371/journal.pone.0181330)
Supplement: S1 Fig — (PDF) [file pone.0181330.s001.pdf]

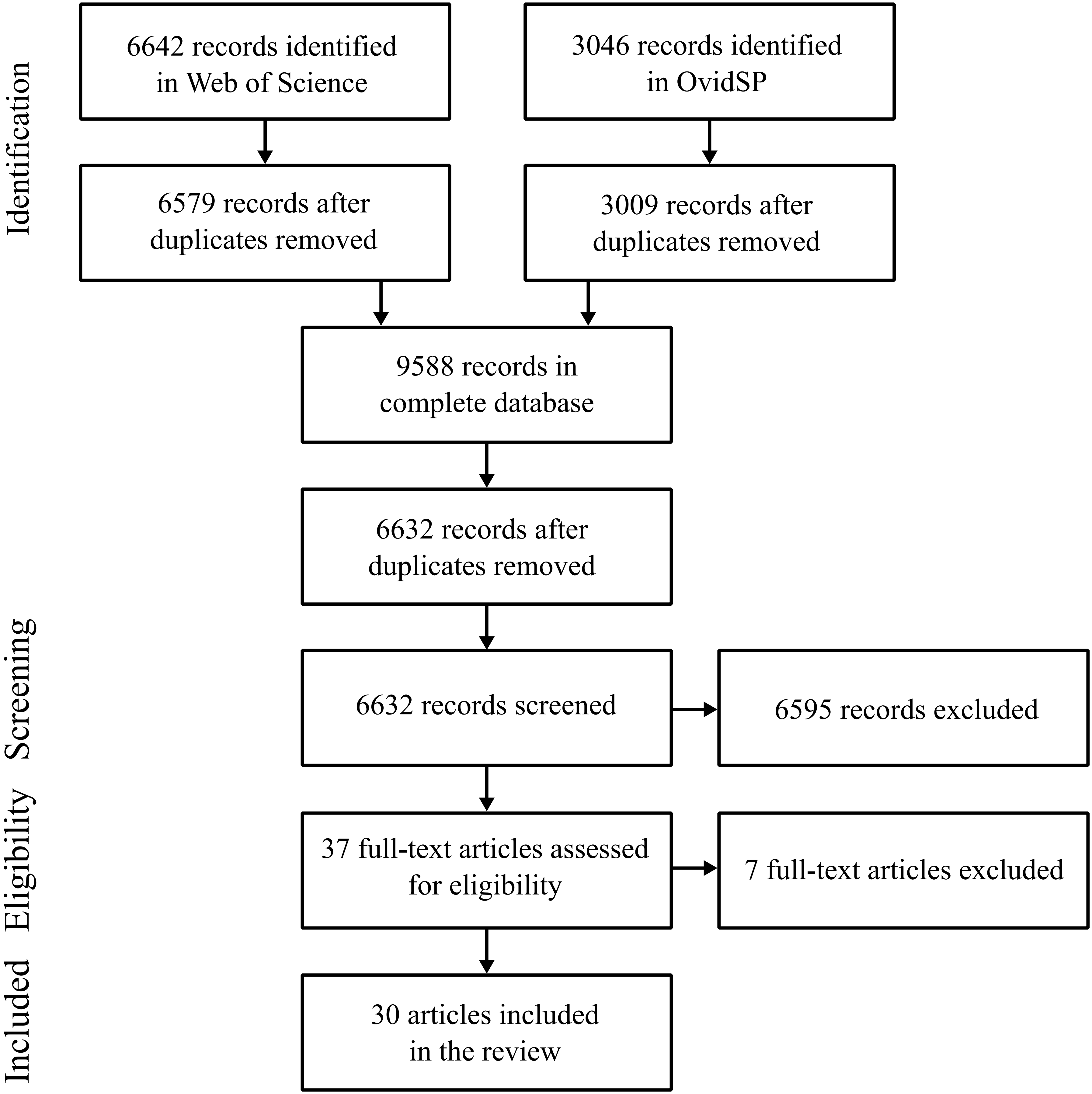
Identification
Screening
Eligibility
Included
6642 records identified in Web of Science
3046 records identified in OvidSP
6579 records after duplicates removed
3009 records after duplicates removed
9588 records in complete database
6632 records after duplicates removed
6632 records screened
6595 records excluded
37 full-text articles assessed for eligibility
7 full-text articles excluded
30 articles included in the review
