# Supplementary material for: Dealing with foreign cultural paradigms: A systematic review on intercultural challenges of international medical graduates
Source: PLoS One. 2017 Jul 17;12(7):e0181330. doi: 10.1371/journal.pone.0181330 (PMC5513557; doi:10.1371/journal.pone.0181330)
Supplement: S1 Text — (PDF) [file pone.0181330.s002.pdf]

S1 Text

Search strategy via Web of Science.

1. oversea* NEAR/3 (train* OR qualifed*)
2. medic* NEAR/3 immigr*
3. (foreign* OR international*) NEAR/3 medical* NEAR/3 graduate*
4. (foreign* OR international*) NEAR/3 doctor*
5. (foreign* OR international*) NEAR/3 physician*
6. 1. OR 2. OR 3. OR 4. OR 5.
