# Supplementary material for: Dealing with foreign cultural paradigms: A systematic review on intercultural challenges of international medical graduates
Source: PLoS One. 2017 Jul 17;12(7):e0181330. doi: 10.1371/journal.pone.0181330 (PMC5513557; doi:10.1371/journal.pone.0181330)
Supplement: S2 Text — (PDF) [file pone.0181330.s003.pdf]

S2 Text

Inclusion criteria.

1. The observed population were physicians working as immigrants in an another country than their country of origin and training
2. The following topics were covered:
   (a) Communication with
      i. Patients and their families
      ii. Other physicians
      iii. Other health professionals
      iv. Specific type of miscommunication
   (b) Attitudes or expectations regarding issues that are important in treating patients or in health care system in general (death, illness, health, truth, delivering bad news etc.)
   (c) Medical documentation and patient presentation
   (d) Patient treatment
      i. Duration
      ii. Type, procedures, medication
   (e) Health care system
      i. Structure
      ii. Economic factors
   (f) Status of the physician
      i. In society
      ii. In the interdisciplinary team
      iii. In the health care system
   (g) Decision-making
      i. Inclusion of the patient
      ii. Inclusion of the family of the patient
      iii. Compliance
   (h) Team structure, capacity for teamwork and acceptance in the team
   (i) Change of self-esteem
   (j) Supervision or support for team inclusion
   (k) Attitude to medical science and teaching
3. Empirical study (qualitative or quantitative)
4. Language: English or German
