# Supplementary material for: Dealing with foreign cultural paradigms: A systematic review on intercultural challenges of international medical graduates
Source: PLoS One. 2017 Jul 17;12(7):e0181330. doi: 10.1371/journal.pone.0181330 (PMC5513557; doi:10.1371/journal.pone.0181330)
Supplement: S3 Text — (PDF) [file pone.0181330.s004.pdf]

S3 Text

Exclusion criteria.

1. The investigated intervention was a cultural competence training.
2. Investigation of intercultural problems, in which the patients and not the physicians are foreign or belonging to a ethnic or cultural minority.
3. Only medical students participated in the study.
