# Supplementary material for: Dealing with foreign cultural paradigms: A systematic review on intercultural challenges of international medical graduates
Source: PLoS One. 2017 Jul 17;12(7):e0181330. doi: 10.1371/journal.pone.0181330 (PMC5513557; doi:10.1371/journal.pone.0181330)
Supplement: S1 Table — (PDF) [file pone.0181330.s006.pdf]

S1 Table

Category system of the qualitative studies.

Main-category	Sub-category
A-1.1 Communication with patients	A-1.1.1 Way of treatment
	A-1.1.2 Duration of treatment
	A-1.1.3 Patient information
	A-1.1.4 Medication
	A-1.1.5 Hierarchy in the physician-patient relationship
	A-1.1.6 Decision-making style
	A-1.1.7 Patient compliance
	A-1.1.8 Emotional support
A-1.2 Communication with relatives	A-1.2.1 Information of the patients family
	A-1.2.2 Involvement of the relatives in decisions
	A-1.2.3 Involvement of the relatives in treatment
	A-1.2.4 Care of the patient
A-1.3 Communication with native physicians	A-1.3.1 Presentation of the patient
	A-1.3.2 Hierarchy among physicians
	A-1.3.3 Supervisor support
A-1.4 Communication with other health professionals	A-1.4.1 Hierarchy in an interdisciplinary team
	A-1.4.2 Allocation of tasks in an interdisciplinary team
A-1.5 Nonverbal communication	
A-1.6 Communication unspecified	
A-2.1 Health care system	A-2.1.1 Patient documentation
	A-2.1.2 Organizational structure
	A-2.1.3 Economic parameters
	A-2.1.4 Legal parameters
A-3.1 Language	A-3.1.1 Comprehension of language
	A-3.1.2 Use of language
	A-3.1.3 Common speech
	A-3.1.4 Medical terminology
	A-3.1.5 Accent or pronunciation
	A-3.1.6 Small talk or humor
A-4.1 Status of physicians	A-4.1.1 In society
	A-4.1.2 In the health care system
	A-4.1.3 In an interdisciplinary team
	A-4.1.4 In the physician-patient relationship
A-5.1 Origin of the IMGs	A-5.1.1 Cultural background
	A-5.1.2 Educational background
A-6.1 Immigration	A-6.1.1 Cultural issues
	A-6.1.2 Organizational issues
	A-6.1.3 Work-related issues
A-7.1 Racism or discrimination	
A-8.1 Gender issues	

Category system of the qualitative studies including the main- and sub-categories.
