# Supplementary material for: Dealing with foreign cultural paradigms: A systematic review on intercultural challenges of international medical graduates
Source: PLoS One. 2017 Jul 17;12(7):e0181330. doi: 10.1371/journal.pone.0181330 (PMC5513557; doi:10.1371/journal.pone.0181330)
Supplement: S2 Table — (PDF) [file pone.0181330.s007.pdf]

## S2 Table

**Category system based on the questions and instruments of the quantitative studies.**

| | |
|---|---|
| B-1.1 Context - work | B-1.1.1 Stress |
| | B-1.1.2 Resources |
| | B-1.1.3 Well-being |
| B-1.2 Context - colleagues/team | B-1.2.1 Peers |
| | B-1.2.2 Supervisors |
| | B-1.2.3 Other health professionals |
| B-1.3 Context - patients/relatives | |
| B-2.1 Communication among physicians | B-2.1.1 Individual communication |
| | B-2.1.2 Communication in a group |
| B-3.1 Health care system | B-3.1.1 Organizational structure |
| | B-3.1.2 Billing system |
| B-4.1 Clinical skills | |

**Category system based on the questions and instruments of the quantitative studies including the main- and sub-categories.**
