# Supplementary material for: Dealing with foreign cultural paradigms: A systematic review on intercultural challenges of international medical graduates
Source: PLoS One. 2017 Jul 17;12(7):e0181330. doi: 10.1371/journal.pone.0181330 (PMC5513557; doi:10.1371/journal.pone.0181330)
Supplement: S3 Table — (PDF) [file pone.0181330.s008.pdf]

S3 Table

Category system based on the textual results of the quantitative studies.

C-1.1 Context - work	C-1.1.1 Stress
	C-1.1.2 Resources
	C-1.1.3 Well-being
C-1.2 Context - colleagues/team	C-1.2.1 Peers
	C-1.2.2 Supervisors
	C-1.2.3 Other health professionals
C-1.3 Context - patients/relatives	
C-2.1 Communication among physicians	C-2.1.1 Individual communication
	C-2.1.2 Communication in a group
C-3.1 Health care system	C-3.1.1 Organizational structure
	C-3.1.2 Billing system
C-4.1 Clinical skills	

Category system based on the textual results of the quantitative studies including the main- and sub-categories.
