# Supplementary material for: Dealing with foreign cultural paradigms: A systematic review on intercultural challenges of international medical graduates
Source: PLoS One. 2017 Jul 17;12(7):e0181330. doi: 10.1371/journal.pone.0181330 (PMC5513557; doi:10.1371/journal.pone.0181330)
Supplement: S4 Table — (PDF) [file pone.0181330.s009.pdf]

S4 Table

Qualitative studies included in the review.

Study	Country of study	Participants	Country of origin	Summary
Chen et al. (2010)	USA	25 IMGs	Sub-Saharan Africa, South Asia, East Asia, Latin America and Middle East.	IMGs experienced 'both overt and subtle forms of workplace bias and discrimination' and recognized 'professional limitations as part of "the deal"'. 'They described challenges in the transition to the culture and practice of medicine in the US'. In addition, they brought 'unique skills and advantages to the workplace'.
Curran et al. (2008)	Canada	19 IMGs	Africa, South Asia, Middle East.	IMGs and Canadian physicians 'felt it was necessary for new IMGs to receive relevant orientation to the Canadian health system. The influence of an IMGs own cultural beliefs on his or her medical practice as well as an understanding of the cultural background and beliefs of the population he or she would be working with were highlighted as being important. Cultural sensitivity emerged as a significant theme and was believed to be something which needed to be fostered throughout health organizations. Orientation to the community and mentoring were also identified as important components of effective orientation.'
Dahm (2011a)	Australia	29 IMGs	Afghanistan, Bangladesh, Burma, China, India, Indonesia, Iran, Pakistan, Russia, Iraq, Macedonia and Sri-Lanka.	IMGs who participated at a communication skills course in Australia were not 'aware of meaning divergences concerning medical terminology'. Many 'IMGs failed to clarify the meaning of semi-technical terms used by patients in role-plays.'
Dahm (2011b)	Australia	33 IMGs	Northeast Asia, Southern Central Asia, Southeast Asia Middle East, Southern and Eastern Europe.	IMGs who participated at a communication skills course in Australia were 'novices in PCC: they framed consultations as interviews as opposed to conversations, maintained topic control instead of allowing digressions, and focused on achieving simple coherence rather than seeing the consultation as a whole.'
Dahm et al. (2015)	Australia	5 IMGs	Southern Central Asia and Southeast Asia.	IMGs in Australia who were videotaped in their patient consultations showed several difficulties in communicating with patient.

Diaz et al. (2011)	Norway	7 IMGs	Eastern Europe, Asia, South America and Africa.	IMGs in Norway 'described a gradual process of becoming bicultural'. They 'described being aware of cultural issues in consultations with immigrant and Norwegian patients, but rarely making these issues explicit'. Furthermore, they 'experienced a big workload related to immigrant patients, but they accepted this as a natural part of their work'. They also 'felt that they had to work harder and be more careful than their Norwegian colleagues in order to avoid complaints from patients and to be accepted by colleagues'.
Dorgan et al. (2009)	USA	12 IMGs	The Caribbean, Colombia, Denmark, Iran, India, Pakistan and Peru.	Foreign physicians in the USA considered their foreign medical education (with a focus on science and a 'lack of communication training'), 'unfamiliar dialects' and 'different rapport-building expectations' as a 'source of problematic communication'.
Fiscella et al. (2000)	USA	10 IMGs and 4 US physicians	Pakistan, India, Taiwan, Costa Rica.	IMGs who worked in the US and their native colleagues talked about their 'struggles for acceptance, fear of rejection, fear of disappointing patients' and their 'struggle to express caring transculturally'.
Gasiorek et al. (2012)	Germany, Belgium, Sweden and Italy	150 IMGs and 54 native physicians	Germany, Poland, Arabia and others.	Foreign physicians who worked in different European countries and their native colleagues agreed that IMGs 'could use additional training related to everyday medical language, fluency, idioms, pronunciation, humor, and local dialects'. 'IMGs generally felt confident in their communication skills (and thought others saw them as competent), but their colleagues reported a number of concerns including difficulty with small talk, nonverbal communication, and observance of (related) local cultural norms'.
Hall et al. (2004)	Canada	16 IMGs, 7 Canadian physicians, 1 nurse, 1 standardized patient, 2 social workers and 3 program directors	Saudi Arabia, Libya, United Arab Emirates and Ireland.	IMGs and Canadian health personnel recommended improvements for the IMGs in the following areas: 'English-language skills', 'how to get things done in the hospital/healthcare system', 'opportunities to practice specific skills', 'adequate support system for IMGs' and 'faculty and staff education on the cultural challenges faced by IMGs'.

Huijskens et al. (2010)	Netherlands	32 IMGs	Iraq, Syria, Iran, Afghanistan, Bulgaria, Rumania, former Yugoslavia, Colombia and Peru.	IMGs who worked in the Netherlands reported 'difficulties in accessing information on complementary medical education and lack of (financial) support. Perseverance was reported to be essential. Financial and social support were also reported as facilitating factors. Lack of command of the Dutch language and age were seen as barriers to securing employment and entrance to specialization.'
Jain et al. (2011)	USA	12 IMGs	India, China, Jordan, Lebanon, Nigeria, Philippines	IMGs who work in the US 'identified three major areas that posed a barrier to communicating effectively with patients namely language, affect related issues, and differences in cultural norms regarding medical interaction'. They used the following 'strategies when interacting with their patients to account for the intercultural and inter-group differences, including repeating information, changing speaking styles, and using nonverbal communication'.
Klingler et al. (2016)	Germany	20 IMGs	Romania, Poland, Russia, Greece, Lybia, Iran and Syria.	IMGs in Germany described 'difficulties relating to healthcare institutions, own competencies, and interpersonal interactions. They experienced certain legal norms, the regulation of licensure and application for work, and the organization of the hospital environment as inadequate. Most struggled with their lack of setting-specific (language, cultural, clinical, and system) knowledge. Furthermore, behavior of patients and co-workers was perceived as discriminating or inadequate for other reasons'.

Legido-Quigley et al. (2015)	UK	23 IMGs	France 1, Ireland, Italy, Germany, Greece, Hungary, Malta, Portugal, Slovenia, Spain and the Netherlands.	IMGs from other European countries working in the UK 'highlight they were well prepared and their main motivation to migrate was to learn new skills and experience a new health care system. Some interviewees reported initially having language problems, but most noted that this was resolved after a few months. These doctors overwhelmingly reported having very positive experiences with patients, enjoying a NHS structure that was less hierarchical structure than in their home systems, and appreciating the emphasis on evidence-based medicine. Interviewees mostly complained about the lack of cleanliness of hospitals and gave some examples of risk to patient safety. Interviewees did not experience discrimination other than some instances of patronizing and snobbish behavior. However, a few believed that their nationality was a block to achieving senior positions. Overall, interviewees reported having enjoyable experiences with patients and appreciating what the NHS had to offer'.
Lockyer et al. (2007)	Canada	19 IMGs	South Africa, South America, Pakistan, Europe, UK and Japan.	IMGs who are adapting to practice in Canada 'described two types of learning: learning associated with studying for Canadian examinations required to remain and practice in the province and learning that was required to succeed at clinical work in a new setting'. The 'second type of learning included regulations and systems, patient expectations, new disease profiles, new medications, new diagnostic procedures, and managing the referral process'.
Lockyer et al. (2010)	Canada	25 IMGs, 9 Canadian physicians and 1 administrator	South Africa, other parts of Africa, India an other parts of Asia, Europe.	'IMGs and medical leaders' in Canada 'recognized that learning and support were needed by physicians without previous experience in Canada'. 'Although medical leaders believed the new information was explicit, readily available, and could be learned from short explanations and lists; IMGs found that guidelines and expectations were implicit, confusing, and contradictory'.
Mahajan et al. (2007)	UK	12 IMGs	India, Pakistan and Sri Lanka.	IMGs in the UK perceived 'lack of information about the National Health Service (NHS)/Royal Colleges, inappropriate communication skills, difficulties in team working, difficulties in preparing for Royal College examinations, visa and job hunting, and social and cultural isolation'.

McDonnel et al. (2008)	Australia	8 IMGs and 7 Australian physicians	Indian subcontinent, Middle East, Eastern Europe and Egypt.	IMGs and medical supervisors in Australia identified 'language and communication' as 'the most common challenges' for IMGs.
McGrath et al. (2011)	Australia	30 IMGs	India, Sri Lanka, Iran, South Africa, Sudan, Pakistan, Caribbean, Russia, Philippines, Indonesia, Egypt, Serbia and Afghanistan.	'Multidisciplinary collaboration is a new concept for IMGs integrating into the Australian healthcare system.'
McGrath et al. (2012)	Australia	30 IMGs	India, Sri Lanka, Iran, South Africa, Sudan, Pakistan, Caribbean, Russia, Philippines, Indonesia, Egypt, Serbia and Afghanistan.	'Understanding patient-centered communication is a major challenge faced by IMGs during integration in the Australian health system. They perceive that this difficulty is associated with the major shift from the culture of their country of origin (described as paternalistic doctor-dominated communication system; standard practice to talk to the family and not the patient) to the very different health care culture of Australia (perceived to be more educated and informed consumers that demand high levels of information and discussion).'
Morrow et al. (2013)	UK	64 IMGs and 12 educational supervisors	Sierra Leone, Nigeria, Egypt, Iraq, United Arab Emirates, Austria, Bangladesh, India, Italy, Lithuania, Malta, Pakistan, Poland, Romania, Russia, Afghanistan, Cuba, Jordan, Sudan and Syria.	IMGs in the UK experienced changes 'in relation to workplace hierarchies and interprofessional relationships', 'ways of interacting' and 'doctor-patient/family relationship'.
Osta et al. (2016)	USA	26 IMGs	Turkey, Pakistan, Venezuela, India, Saudi Arabia , Haiti Mexico, Lithuania, Syria, Colombia, Philippines and Peru.	IMGs in the USA showed a need of 'understanding the education system and family structure, social determinants of health, communication with African American parents, contraception, physician handoffs, physicians role in prevention, adolescent health, and physicians role in child advocacy'.
Rao et al. (2013)	USA	11 IMGs and 7 US physicians	India, Nepal, Jordan, Peru, Nigeria and Saudi Arabia.	'IMGs confronted work-related and adjustment challenges during post-graduate year one in the U.S. that included practicing medicine in the U.S., communicating with medical supervisors and nursing staff, communicating with patients, and relocating to the U.S.' American physicians 'confronted work-related challenges that included medical decision-making and medical licensing'.

Searight et al (2006)	USA	10 IMGs	India, Macedonia, Bosnia-Herzegovina, The Philippines, Egypt and Iraq.	IMGs 'described many unique challenges to adjusting to health care, medical education, and the "rules" of medical practice in the United States. Documentation and charting formats were often seen as novel and confusing. Many residents were unclear about how to interact with support staff'. They described many differences 'with regard to the physician-patient relationship'.
Skjeggestad et al. (2017)	Norway	16 IMGs and 12 Norwegian physicians and nurses	Scandinavia, EU, Russia, South and Central America, Asia, Africa, USA and Australia.	Norwegian doctors and nurses as well as IMGs 'experienced that language barriers caused difficulties in their everyday collaboration. Furthermore, the participants descriptions of language barriers encompassed a wide range of topics, including semantics (e.g., specialized professional vocabulary, system knowledge), pragmatics (e.g., using language in doctor-patient and interprofessional interactions), and specific culturally sensitive topics'.
Slowther et al. (2012)	UK	128 IMGs	Pakistan, Nigeria, India, Italy, Greece, South Africa, Hungary, Iran, Poland, Egypt, Russia, Spain, Germany, United States, Madagascar, Bangladesh, Afghanistan, Romania, Somalia, China and Iraq.	'Non-UK qualified doctors reported clear differences in the ethical and legal framework for practicing medicine between the UK and their country of qualification, particularly in the model of the doctor-patient relationship. The degree of support for non-UK-qualified doctors in dealing with ethical concerns is related to the type of post they work in. European doctors describe similar difficulties with working in an unfamiliar regulatory framework to their non-European colleagues.'
Sockalingam et al. (2014)	Canada	53 IMGs and 27 Canadian physicians	Middle East, Central/South America, United States, Europe, Asia, Africa, and Oceania.	IMGs in Canada 'reported the greatest difficulty with adapting to the hospital system, medical documentation, and balancing ones professional and personal life.' They talked about 'disorientation, disconnection, interprofessional team challenges, a need for IMG fellow resources, and a benefit from training in a multicultural setting.' Their 'supervisors believed that fellows had the greatest difficulty with managing language and slang in Canada, the healthcare system, and an interprofessional team.'

Sommer et al. (2012)	Australia	5 IMGs and 2 Australian physicians	Nigeria, Egypt, United Kingdom, India and Singapore.	'IMG-patient communication barriers were considered significant' in a rural Australian region 'as identified by both IMG and Australian physicians'. The native physicians 'indicated they were aware of IMG-patient communication issues resulting in subsequent consults with patients to explain results and diagnoses. Significantly, a lack of communication between' Australian doctors 'and IMG also emerged, creating a further barrier to effective communication'.
Terry et al. (2014)	Australia	105 IMGs	India, Sri Lanka, Iran, Myanmar, South Africa, Malaysia, Pakistan, the Philippines, Vietnam, Mexico, Lebanon, and other countries.	IMGs 'are satisfied in their current employment. However, interview participants indicated there were a number of barriers to practising medicine in' Australia 'as well as factors that would influence ongoing employment'. 'Despite these challenges, professional support was recognised as a key contributor to professional satisfaction, particularly among IMGs who had just arrived'.
Teodorescu et al. (2013)	Romania	22 Romanian physicians working as IMGs in other European countries.	Romania	Romanian doctors who worked in different European countries felt that their 'experience abroad was the opportunity to develop interaction skills with patients. Practicing in other health systems meant changing attitude on the doctor-patient relationship'.
Triscott et al. (2016)	Canada	13 allied health professionals, 12 Canadian physicians, 2 IMGs	Unknown.	Canadian and international medical graduates as well as allied health care professionals 'identified that IMG residents brought multiple strengths to Canadian practice including strong clinical knowledge and experience, high education level, the richness of varied cultural perspectives, and positive personal strengths. At the same time, IMG residents appeared to experience challenges in the areas of: (1) communication skills', '(2) clinical practice', '(3) learning challenges', '(4) cultural differences' and (5) personal struggles'.
Verma et al. (2016)	UK	10 IMGs	Pakistan, India, Egypt, Myanmar, Malaysia and Indonesia.	'The majority of communication failures' of IMGs accounted 'in station 2, "history taking" and station 4, "communication skills and ethics" of the MRCP(UK) PACES examination. Two themes, the ability to detect clues and the ability to address concerns, related directly to the overall construct managing patients' concerns. Three other themes were found to impact the whole consultation. These were building relationships, providing structure and explanation and planning.

Warwick et al. (2014)	UK	12 IMGs	Nigeria, Germany, Pakistan, India, Bangladesh, Philippines and Romania.	IMGs in the UK reported difficulties in 'transition into the culture of the' National Health Service 'and UK general practice'. 'Patient-centered care' and 'language' constituted additional problems.
Woodward-Kron et al. (2015)	Australia	48 IMGs	India, Iran, China and other countries.	IMGs in Australia 'demonstrated aspects of patient-centred interviewing but were hindered by limited interactional competence to elicit information and negotiate behaviours as well as a limited repertoire of English grammar, vocabulary, and phonological phrasing for effective interaction'.
Yates et al. (2016)	Australia	1 IMG and 1 Australian physician	Unknown.	Compared to an Australian Medical Graduate an IMG showed deficits in communicating with a nurse.

Overview about the qualitative ($n = 31$) and mixed methods studies ($n = 4$; $n_{\mathbf{sum}} = 35$) including the country of origin of the IMGs. If possible, the text of the summary was kept in its original form.
