# Supplementary material for: Dealing with foreign cultural paradigms: A systematic review on intercultural challenges of international medical graduates
Source: PLoS One. 2017 Jul 17;12(7):e0181330. doi: 10.1371/journal.pone.0181330 (PMC5513557; doi:10.1371/journal.pone.0181330)
Supplement: S5 Table — (PDF) [file pone.0181330.s010.pdf]

S5 Table

Quantitative studies included in the review.

Study	Country of study	Participants	Country of origin	Summary
Aalto et al. (2014)	Finland	3646 Finnish physicians and 553 IMGs	Russia/former Soviet Union, Estonia, other EU-countries and other countries.	IMGs in Finland 'worked more often in primary care and on-call services and less often in leadership positions than' native physicians. 'They more often experienced lack of professional support and lower work-related well-being compared with native' physicians. Those IMGs 'who had lived for a shorter time in Finland perceived less stress related to electronic patient records systems and higher organizational justice compared with native physicians or those foreign physicians who had migrated earlier'.
Fernandez-Pol et al. (1989)	USA	113 IMGs and 36 USMGs	Unknown.	In comparison to the IMGs, the American physicians 'tend to be less authoritarian-restrictive inclined, tend to report less adherence toward an unsophisticated benevolent approach, and less adherence toward an interpersonal-etiology approach'. Both 'showed similar adherence to the concepts of the mental hygiene movement'.
Harding et al. (2010)	Australia	1127 patients of 5 IMGs and 5 Australian physicians	Unknown.	Australian Patients, who evaluated the consultation with their General Practitioner (GP), 'had high levels of satisfaction with their GPs, irrespective of whether the GP was an IMG or not'.
Kuusio et al. (2013)	Finland	656 Finnish physicians and 176 IMGs	Russia, Estonia, other countries.	'Intention to leave was more common among' IMG General Practitioners (GPs) 'than among Finnish GPs'. 'High job demands were associated with higher intention to leave from primary care both in foreign-born (...) and Finnish GPs (...). 'Lack of job control, patient-related stress, and stresses related to teamwork were associated with higher intention to leave only among Finnish GPs'.
Kwon et al. (1984)	USA	171 US physicians, 45 IMGs	Unknown.	The attitude of IMGs and American physicians 'toward patient management' was 'different in three controversial areas: consumer benefit, inclusion of physicians fee into the prospective payment system, and quality of care'.
Lillis et al. (2014)	New Zealand	353 IMGs	Unknown.	'The overall number of IMGs with one or more unsatisfactory reports was 37 (10.4%)''during their first year of supervised clinical practice in New Zealand'. 'When unsatisfactory reports are generated, the most common concerns are in the area of 'Clinical judgment', but 'Clinical Knowledge and Skills' as well as 'Patient Communication' issues were almost as common'.

Myerholz (2014)	USA	1703 patients of 75 IMGs and 45 US physicians	Unknown.	The interpersonal and communication skills of US medical graduates in a family medicine residency program were rated significantly better by their patients than those of their international colleagues.
Narasimhan et al. (2006)	New Zealand	51 New Zealands doctors, 17 IMGs and 58 nurses	Unknown.	New Zealand hospital staff rated the following areas of IMGs unsatisfactory: 'clinical documentation; communication with patients, families, and other health professionals; knowledge of hospital policies and procedures and medicolegal matters; and some aspects of patient management. There was no difference in median ratings between doctors and nurses'.
Pantenburg et al. (2016)	Germany	1701 German physicians and 192 IMGs	Czech Republic, Slovakia, Poland, Austria, Romania, Hungary, Ukraine, Syria, Bulgaria, Greece, Russia, Lithuania, Bosnia, France, Italy, Moldova, Azerbaijan, Belarus, Georgian Republic, India, Indonesia, Kosovo, Latvia, Lebanon, Luxemburg, Macedonia, Mexico, Serbia, Switzerland, Turkey and other countries not specified.	IMGs in Germany 'were more satisfied with aspects related to patient care, such as "possibility to treat patients as you deem optimal" and "relationship with patients"' compared to their German colleagues. 'However, they were less satisfied with aspects related to human relations, such as "work atmosphere", relationship with co-workers, and "social status". Foreign-national physicians were also less satisfied with the aspect "work enjoyment"'.
Rolfe et al. (1994)	Australia	382 Australian physicians, 55 New Zealand doctors and 44 IMGs	Unknown.	Australian graduates 'were rated more favorably' by their supervisors than IMGs. In addition, 'women were rated better than men, and younger interns better than older interns'.
Sullivan et al. (2001)	USA	337 patients, 45 US physicians, 27 IMGs and 66 nurses	Unknown.	'Physicians and nurses' in an American hospital 'underestimate the number of patients who want detailed information'. IMGs 'appeared to be more likely to change their criteria for informing patients and, along with American-educated nurses, were more willing to participate in formal discussions of the issue'.

Zulla et al. (2008)	Canada	87 IMGs, 45 Program directors	Unknown.	'IMGs and Program Directors' in Canada 'differ in their perspectives as to what are considered challenges to foreign-trained physicians entering residency training. Both groups agree that an orientation program is necessary for incoming IMGs prior to starting their residency program'.

Overview about the quantitative ($n = 12$) studies including the country of origin of the IMGs. If possible, the text in the summary was kept in its original form.
