# Supplementary material for: Dealing with foreign cultural paradigms: A systematic review on intercultural challenges of international medical graduates
Source: PLoS One. 2017 Jul 17;12(7):e0181330. doi: 10.1371/journal.pone.0181330 (PMC5513557; doi:10.1371/journal.pone.0181330)
Supplement: S6 Table — (PDF) [file pone.0181330.s011.pdf]

S6 Table

Results of the qualitative studies.

Category	T-1	T-2	T-3	T-4	Total
A-1.1 Communication with patients	[11, 12, 14, 17, 23, 25, 28, 29, 31, 32]	[8, 10]	[6, 14, 30]	[12, 14, 29, 30]	19
A-1.1.1 Way of treatment	[3, 5, 7, 12, 20, 24, 27, 31, 32]	[33]	[8, 17, 18, 24, 30, 33]	1 [34]	17
A-1.1.2 Duration of treatment	0	0	[20, 30]	0	2
A-1.1.3 Patient information	[31]	0	[14, 20, 21, 30]	[14]	6
A-1.1.4 Medication	[10]	0	[14]	[14]	3
A-1.1.5 Hierarchy in the physician-patient relationship	[7, 12, 24, 25, 31, 32]	[16]	[7, 14, 16, 20, 21, 24, 26, 30]	[14, 30]	17
A-1.1.6 Decision-making style	[13, 17, 23]	0	[1, 20–22, 24]	0	8
A-1.1.7 Patient compliance	0	0	[30]	[30]	2
A-1.1.8 Emotional support	[5, 11, 34]	0	0	[12]	4
A-1.2 Communication with relatives	[17]	0	[8, 20, 30]	0	4
A-1.2.1 Information of the patient's family	[25, 31]	0	[8, 12, 18, 21, 26]	0	7
A-1.2.2 Involvement of the relatives in decisions	0	0	[8]	0	1
A-1.2.3 Involvement of the relatives in treatment	0	0	[8, 21]	0	2
A-1.2.4 Care of the patient	0	0	[19]	0	1
A-1.3 Communication with native physicians	[10, 14, 17, 28, 29]	0	[6, 8, 30]	[10, 29, 30]	11
A-1.3.1 Presentation of patients	[10]	0	[22]	0	2
A-1.3.2 Hierarchy among physicians	[10, 23, 31]	0	[14, 21, 23]	0	6
A-1.3.3 Supervisor support	[9, 17, 31]	0	[8, 22]	[16, 29]	7
A-1.4 Communication with other health professionals	[13, 14, 17, 23, 25, 32]	0	0	0	6
A-1.4.1 Hierarchy in an interdisciplinary team	[8–10, 31]	0	[8, 9, 21, 24]	[14]	9
A-1.4.2 Allocation of tasks in an interdisciplinary team	[9, 13, 17]	[16, 19]	[8, 19]	0	7
A-1.5 Nonverbal Communication	[18, 26, 31, 32]	[8, 10]	[21]	[12]	8
A-1.6 Communication unspecified	[17, 18, 25, 31]	[2, 17]	0	[10, 18]	8
A-2.1 Health care system	[18, 25, 27, 29, 31]	[14]	0	[14]	7
A-2.1.1 Patient documentation	[13–15, 24]	[15, 16]	[22]	[22]	8
A-2.1.2 Organizational structure	[1, 13, 15, 19, 23, 24, 31, 33]	[2, 8, 9, 15, 16]	[1, 26, 30]	0	16
A-2.1.3 Economic parameters	[14, 15, 23]	[15, 16]	[30]	0	6
A-2.1.4 Legal parameters	[13, 24, 27, 31]	[8]	[1, 12, 18, 26]	0	9
A-3.1 Language	[9, 10, 14, 18, 23, 25, 26, 29, 31, 33]	[2]	0	[1, 6, 18]	14
A-3.1.1 Comprehension of language	[13]	[8]	0	[17]	3
A-3.1.2 Use of language	[5, 10, 12, 13, 22, 31, 34]	[8, 10]	0	[17]	10
A-3.1.3 Common speech	[1, 4, 7, 10, 13, 18, 22, 25, 28, 33]	[8, 10]	[4]	0	13
A-3.1.4 Medical terminology	[4, 5, 10, 12, 13, 31, 32]	[8, 10]	[4]	0	10
A-3.1.5 Accent or pronunciation	[7, 10, 12, 17, 18, 22, 23, 25, 28, 31, 34]	[10]	0	0	12
A-3.1.6 Small talk or humor	[5, 10, 12, 22, 31]	0	0	0	5
A-4.1 Status of physicians	0	0	0	0	0
A-4.1.1 In society	[7, 31]	0	[1, 18, 20, 21]	0	6
A-4.1.2 In the health care system	[13]	0	[21, 26, 30]	0	4
A-4.1.3 In an interdisciplinary team	[8, 9, 31]	0	[8, 9, 21, 24, 27]	0	8
A-4.1.4 In the physician-patient relationship	[7, 12, 24, 25]	[16]	[7, 16, 20, 21, 24, 26, 30]	0	12
A-5.1 Origin of the IMGs	[13]	0	0	0	1
A-5.1.1 Cultural background	[28, 31]	0	[11, 21, 24]	[31]	6
A-5.1.2 Educational background	[7, 13, 31]	[8]	[7, 21, 24]	[1, 31]	9
A-6.1 Immigration	0	0	0	[14]	1
A-6.1.1 Cultural issues	[8, 10, 13, 17, 18, 22, 27, 31, 33]	[2, 10]	[6, 8, 24]	[1, 6, 8, 27]	18
A-6.1.2 Organizational issues	[9, 13, 14, 17, 18, 27, 29, 31]	[2, 8, 27]	[24]	[9, 14, 17, 29, 31]	17
A-6.1.3 Work-related issues	[1, 9, 13, 14, 17, 23, 31, 33]	[17, 27]	[6, 16, 18, 22, 24, 29]	[24]	17
A-7.1 Racism or discrimination	[13, 14, 18, 23]	0	0	[14]	5
A-8.1 Gender issues	[13, 22, 31]	0	[11]	0	4

Results of the the qualitative ($n = 31$) and mixed methods studies ($n = 4$; $n_{\textbf{sum}} = 35$) including the main- and sub-categories. Assessment-types: T-1 = Problem, T-2 = Improvement opportunity, T-3 = Difference, T-4 = Positive judgment/attitude.

References

1. Chen PGC, Nunez-Smith M, Bernheim SM, Berg D, Gozu A, Curry LA. Professional experiences of international medical graduates practicing primary care in the United States. Journal of General Internal Medicine. 2010;25(9):947–953.

2. Curran V, Hollett A, Hann S, Bradbury C. A qualitative study of the international medical graduate and the orientation process. Canadian Journal of Rural Medicine: the Official Journal of the Society of Rural Physicians of Canada: 2008;13(4):163–169.

3. Dahm MR. Patient centred care: Are international medical graduates' expert novices'? Australian Family Physician. 2011;40(11):895.

4. Dahm MR. Exploring perception and use of everyday language and medical terminology among international medical graduates in a medical ESP course in Australia. English for Specific Purposes. 2011;30(3):186–197.

5. Dahm MR, Yates L, Ogden K, Rooney K, Sheldon B. Enhancing international medical graduates' communication: the contribution of applied linguistics. Medical Education. 2015;49(8):828–837.

6. Díaz E, Hjörleifsson S. Immigrant general practitioners in Norway: a special resource? A qualitative study. Scandinavian Journal of Public Health. 2011;39(3):239–244.

7. Dorgan KA, Lang F, Floyd M, Kemp E. International medical graduate-patient communication: a qualitative analysis of perceived barriers. Academic Medicine. 2009;84(11):1567–1575.

8. Hall P, Keely E, Dojeiji S, Byszewski A, Marks M. Communication skills, cultural challenges and individual support: challenges of international medical graduates in a Canadian healthcare environment. Medical Teacher. 2004;26(2):120–125.

9. Huijskens EG, Hooshiaran A, Scherpbier A, Van Der Horst F. Barriers and facilitating factors in the professional careers of international medical graduates. Medical Education. 2010;44(8):795–804.

10. Gasiorek J, van de Poel K. Divergent perspectives on language-discordant mobile medical professionals' communication with colleagues: an exploratory study. Journal of Applied Communication Research. 2012;40(4):368–383.

11. Fiscella K, Roman-Diaz M, Lue BH, Botelho R, Frankel R. 'Being a foreigner, I may be punished if I make a small mistake': assessing transcultural experiences in caring for patients. Family Practice. 1997;14(2):112–116.

12. Jain P, Krieger JL. Moving beyond the language barrier: The communication strategies used by international medical graduates in intercultural medical encounters. Patient Education and Counseling. 2011;84(1):98–104.

13. Klingler C, Marckmann G. Difficulties experienced by migrant physicians working in German hospitals: a qualitative interview study. Human Resources for Health. 2016;14(1):57.

14. Legido-Quigley H, Saliba V, McKee M. Exploring the experiences of EU qualified doctors working in the United Kingdom: A qualitative study. Health Policy. 2015;119(4):494–502.

15. Lockyer J, Fidler H, de Gara C, Keefe J. Learning to practice in Canada: the hidden curriculum of international medical graduates. Journal of Continuing Education in the Health Professions. 2010;30(1):37–43.

16. Lockyer J, Hofmeister M, Crutcher R, Klein D, Fidler H. International medical graduates: learning for practice in Alberta, Canada. Journal of Continuing Education in the Health Professions. 2007;27(3):157–163.

17. Mahajan J, Stark P. Barriers to education of overseas doctors in paediatrics: a qualitative study in South Yorkshire. Archives of Disease in Childhood. 2007;92(3):219–223.

18. McDonnell LB, et al. International medical graduates: Challenges faced in the Australian training program. Australian Family Physician. 2008;37(6):481.

19. McGrath PD, Henderson D, Tamargo J, Holewa HA. 'All these allied health professionals and you're not really sure when you use them': insights from Australian international medical graduates on working with allied health. Australian Health Review. 2011;35(4):418–423.

20. McGrath P, Henderson D, Tamargo J, Holewa H, et al. Doctor-patient communication issues for international medical graduates: research findings from Australia. Education for Health. 2012;25(1):48.

21. Morrow G, Rothwell C, Burford B, Illing J. Cultural dimensions in the transition of overseas medical graduates to the UK workplace. Medical Teacher. 2013;35(10):e1537–e1545.

22. Osta AD, Barnes MM, Pessagno R, Schwartz A, Hirshfield LE. Acculturation needs of pediatric international medical graduates: A qualitative study. Teaching and Learning in Medicine. 2016; p. 1–10.

23. Rao A, Freed CR, Trimm RF. International and American medical graduates in a US pediatric residency program: A qualitative study about challenges during post-graduate year 1. Medical Teacher. 2013;35(10):815–819.

24. Searight HR, Gafford J. Behavioral science education and the international medical graduate. Academic Medicine. 2006;81(2):164–170.

25. Skjeggestad E, Gerwing J, Gulbrandsen P. Language barriers and professional identity: A qualitative interview study of newly employed international medical doctors and Norwegian colleagues. Patient Education and Counseling. 2017; 100 (8): 1466 – 1472.

26. Slowther A, Hundt GL, Purkis J, Taylor R. Experiences of non-UK-qualified doctors working within the UK regulatory framework: a qualitative study. Journal of the Royal Society of Medicine. 2012;105(4):157–165.

27. Sockalingam S, Khan A, Tan A, Hawa R, Abbey S, Jackson T, et al. A framework for understanding international medical graduate challenges during transition into fellowship programs. Teaching and Learning in Medicine. 2014;26(4):401–408.

28. Sommer J, Macdonald W, Bulsara C, Lim D. Grunt language versus accent: the perceived communication barriers between international medical graduates and patients in Central Wheatbelt catchments. Australian Journal of Primary Health. 2012;18(3):197–203.

29. Terry DR, Lê Q, Hoang H. Satisfaction amid professional challenges: International medical graduates in rural Tasmania. The Australasian Medical Journal. 2014;7(12):500.

30. Teodorescu C, Manea T, Gavrilovici C, Oprea L. International doctor migration and the doctor-patient relationship. Revista Romana de Bioetica. 2013;11(2).

31. Triscott JA, Szafran O, Waugh EH, Torti JM, Barton M. Cultural transition of international medical graduate residents into family practice in Canada. International Journal of Medical Education. 2016;7:132.

32. Verma A, Griffin A, Dacre J, Elder A. Exploring cultural and linguistic influences on clinical communication skills: a qualitative study of International Medical Graduates. BMC Medical Education. 2016;16(1):162.

33. Warwick C. How international medical graduates view their learning needs for UK GP training. Education for Primary Care. 2014;25(2):84–90.

34. Woodward-Kron R, Fraser C, Pill J, Flynn E. How we developed doctors speak up: an evidence-based language and communication skills open access resource for international medical graduates. Medical Teacher. 2015;37(1):31–33.

35. Yates L, Dahm MR, Roger P, Cartmill J. Developing rapport in interprofessional communication: Insights for international medical graduates. English for Specific Purposes. 2016;42:104–116.
